# Combining SDS-PAGE to capillary zone electrophoresis-tandem mass spectrometry for high-resolution top-down proteomics analysis of intact histone proteoforms

**DOI:** 10.1002/pmic.202300650

**Published:** 2024-07-17

**Authors:** Fei Fang, Guangyao Gao, Qianyi Wang, Qianjie Wang, Liangliang Sun

**Affiliations:** Department of Chemistry, Michigan State, University, East Lansing, Michigan, USA

**Keywords:** capillary electrophoresis-mass spectrometry, histone proteoform, post-translational modifications, SDS-PAGE, top-down proteomics

## Abstract

Mass spectrometry (MS)-based top-down proteomics (TDP) analysis of histone proteoforms provides critical information about combinatorial post-translational modifications (PTMs), which is vital for pursuing a better understanding of epigenetic regulation of gene expression. It requires high-resolution separations of histone proteoforms before MS and tandem MS (MS/MS) analysis. In this work, for the first time, we combined SDS-PAGE-based protein fractionation (passively eluting proteins from polyacrylamide gels as intact species for mass spectrometry, PEPPI-MS) with capillary zone electrophoresis (CZE)-MS/MS for high-resolution characterization of histone proteoforms. We systematically studied the histone proteoform extraction from SDS-PAGE gel and follow-up cleanup as well as CZE-MS/MS, to determine an optimal procedure. The optimal procedure showed reproducible and high-resolution separation and characterization of histone proteoforms. SDS-PAGE separated histone proteins (H1, H2, H3, and H4) based on their molecular weight and CZE provided additional separations of proteoforms of each histone protein based on their electrophoretic mobility, which was affected by PTMs, for example, acetylation and phosphorylation. Using the technique, we identified over 200 histone proteoforms from a commercial calf thymus histone sample with good reproducibility. The orthogonal and high-resolution separations of SDS-PAGE and CZE made our technique attractive for the delineation of histone proteoforms extracted from complex biological systems.

## 1 ∣ INTRODUCTION

The nucleosome, playing a key role in the regulation of gene expression, is composed of a DNA molecule wrapped around a histone octamer with H3-H4 and H2A-H2B histone pairs [1, 2]. Histone proteins exert epigenetic regulation via their post-translational modifications (PTMs), including methylation, phosphorylation, acetylation, ubiquitylation, SUMOylation, glycosylation, and ADP-ribosylation, to influence the chromatin structure and recruit chromatin-binding proteins [3, 4]. With the histone PTMs rarely function independently and often found in staggering number of combinatorial patterns, bottom-up proteomics is challenging to reveal the connectivity between co-occurring histone PTMs [5, 6]. Mass spectrometry (MS)-based top-down proteomics (TDP) characterizes intact histone proteoforms and has shown its value for measuring the complex histone proteoforms and discovering combinatorial PTMs of histones [7-9].

Due to the increased diversity from the histone variants and their heavily decorated PTMs, high-resolution separations of different histone proteins (H2A, H2B, H3, H4) before MS measurement is critical for better sensitivity and coverage. The main prevailing methods used for histone proteoform separation are chromatographic separation methods, such as reverse-phase liquid chromatography (LC) [10-13] and weak cation exchange hydrophilic interaction liquid chromatography (WCX-HILIC) [14, 15]. More recently, capillary zone electrophoresis (CZE)-MS has been suggested as a useful tool for TDP analysis of histone proteoforms [16, 17]. CZE separates proteoforms according to their electrophoretic mobility, which is sensitive to the change in charge of proteoforms due to PTMs, for example, acetylation and phosphorylation. Therefore, CZE-MS is a potentially valuable technique for characterizing histone proteoforms. For example, we identified hundreds of histone proteoforms from a commercial calf thymus histone sample via coupling size exclusion chromatography (SEC) or high field asymmetric ion mobility spectrometry (FAIMS) to CZE-MS/MS [16, 17]. Multidimensional separations prior to MS and MS/MS analysis are essential for deciphering the diverse and dynamic histone proteoforms. Although the previous multidimensional LC or electrophoretic methods have shown promising results for separations of histone proteoforms, continuous improvement is still needed due to their extremely high complexity and critically important biological functions.

Sodium dodecyl-sulfate polyacrylamide gel electrophoresis (SDS-PAGE) is one of the most widely used techniques for protein-related biological research, offering high-resolution separation of proteins according to their mass [18]. Recently, Takemori’s group developed Passively Eluting Proteins from Polyacrylamide gels as Intact species for MS (PEPPI-MS) for TDP [19]. The PEPPI-MS method employed SDS-PAGE for protein separation based on their mass and then cut the gel into different pieces to fractionate a complex protein mixture, followed by passive protein extraction from the crushed gel, protein cleanup, and MS and MS/MS analysis. The PEPPI-MS method has been successfully applied to TDP analysis of various complex biological samples (i.e., Escherichia *coli,* human cell lysate, and human serum), showing the potential of the technique for global qualitative and quantitative TDP profiling of complex samples [20-22].

To our best knowledge, the PEPPI-MS method has not been systematically evaluated for TDP analysis of histone proteoforms. In this work, for the first time, we carefully studied the PEPPI-MS method for histone proteoform fractionation based on their mass and combined it with the optimized CZE-MS/MS for high-resolution characterization of histone proteoforms.

## 2 ∣ MATERIALS AND METHODS

### 2.1 ∣ Material and regents

Histone extract (from calf thymus, Product No. 10223565001) and all chemical reagents were obtained from Sigma-Aldrich (St. Louis, MO) unless stated otherwise. LC-MS grade solvents, including water, acetonitrile (ACN), methanol, formic acid (FA) and acetic acid (AA), and hydrofluoric acid (HF) were purchased from Fisher Scientific (Pittsburgh, PA). Acrylamide was purchased from Acros Organics (NJ, USA). Fused silica capillaries (50 μm id/360 μm od) were purchased from Polymicro Technologies (Phoenix, AZ). The Micro Bio-Spin^™^ P-6 Gel Columns (Product No. 7326221) were obtained from BIO-RAD (Hercules, CA),

### 2.2 ∣ Histone protein precipitation with different methods

A total of 6 μg of calf thymus histone protein were dissolved with 100 μL of 0.1% sodium dodecyl sulfate (SDS) or 0.1% n-dodecyl-*β*-maltoside (DDM) in 50 mM ammonium bicarbonate (NH_4_HCO_3_, pH 9). Then, the samples were respectively purified with different published methods with slight modifications, including trichloroacetic acid (TCA) precipitation [23], acetone precipitation [24, 25], chloroform/methanol/water (C/M/W) precipitation [26], and micro-Bio-Spin size exclusion column method. For the micro-Bio-Spin size exclusion column method, 6 μg of calf thymus histone protein was dissolved with 40 μL of 0.1% DDM in 50 mM NH_4_HCO_3_, pH 9, and purified according to the product manual with slight modifications.

For the TCA precipitation method, 33 μL of 100% TCA was added to the 100 μL of sample drop by drop. With the tube inverted several times, the protein sample was incubated on ice for 30 min and collected by centrifugation at 10,000 *g* for 10 min at 4°C. The supernatant was carefully removed, and the pellet was washed with 500 μL of ice-cold acetone. The sample was centrifuged at 16,000 *g* for 5 min at 4°C. The acetone wash and centrifugation steps were repeated. The supernatant was carefully removed, with histone pellet air-dried for 20 min at room temperature. The obtained histone pellet was dissolved with 100 μL of LC-MS grade H_2_O and lyophilized with a vacuum concentrator equipped with a cold trap (Thermo Fisher Scientific).

For the acetone precipitation method, the 100 μL of the sample were vacuum dried to ~30 μL. Then, 120 μL of cold acetone was added to the sample. With the tube inverted several times, the protein sample was incubated on ice for 4 h and collected by centrifugation at 16,000 *g* for 10 min at 4°C. The supernatant was carefully removed, and the sample was subjected to acetone wash followed by a vacuum dry step as described above.

For the rapid acetone precipitation method, the 100 μL of the samples were vacuum dried to ~30 μL. Then, 120 μL of room-temperature acetone was added to the sample. With the tube inverted several times, the protein sample was incubated at room temperature for 2 min and collected by centrifugation at 16,000 *g* for 10 min at room temperature. The supernatant was carefully removed, and the sample was subjected to acetone wash followed by a vacuum dry step as described above.

For the C/M/W precipitation method, 400 μL of methanol, 100 μL of chloroform, and 300 μL of water were in sequence added to the sample and vortexed thoroughly. Then, the sample was centrifuged at 14,000 *g* for 2 min at 4°C, with the top aqueous layer carefully removed. After that, 400 μL of methanol was added and vortexed thoroughly. The sample was centrifuged at 20,000 *g* for 6 min at 4°C. The methanol was carefully removed as much as possible, and the sample was subjected to acetone wash followed by a vacuum dry step as described above.

Micro-Bio-Spin size exclusion column method is a commonly used strategy to remove nonionic detergent [27]. Therefore, the histone sample dissolved in 0.1% DDM was processed according to the product manual with slight modifications. Briefly, the column was inverted several times to resuspend the settled gel and remove any bubbles. Then, snap off the tip and place the column in a 2.0 mL microcentrifuge tube. Centrifuge the column at 1000 *g* for 2 min at 4°C to remove the remaining packing buffer. Apply 500 μL of H_2_O to the column and centrifuge the column at 1000 *g* for 1 min at 4°C to remove the buffer. Discard buffer from the collection tube. Repeat the wash step three times. Put the column in a clean 1.5 mL microcentrifuge tube. Carefully apply the sample directly to the center of the column. After loading the sample, centrifuge the column at 1000 *g* for 4 min at 4°C. The sample was collected and dried with vacuum speed.

### 2.3 ∣ Histone protein separation with SDS-PAGE and extraction

The calf thymus histone protein was separated with home-made SDS-PAGE gel containing an 18% separating gel and a 4% stacking gel. The protein bands corresponding to each histone protein were excised from gels with a razor blade and processed by PEPPI-MS method with modifications [19]. Briefly, the gel pieces were transferred to a disposable tube containing 100 μL of 0.1% SDS (w/v) in 50 mM NH_4_HCO_3_ (pH 9). To extract the histone protein from the gel, the excised gel segments were uniformly ground using a plastic pestle, followed by vigorous shaking at room temperature for 10 min. After filtration through a 0.45 μm cellulose acetate membrane within a Spin-X centrifuge tube filter (Corning, NY), 50 μL of 0.1% SDS (w/v) was added to the filter and the elute was collected and combined. Then, each fraction was processed with the TCA precipitation protocol. Eventually, six fractions were dissolved with sample buffer (50 mM ammonium acetate (NH_4_Ac), adjusted to pH 9 with NH_3_•H_2_O) and subjected to CZE-MS/MS analysis, respectively.

### 2.4 ∣ Capillary coating

According to previous publications [28], a 1-m-long fused silica capillary (50 μm i.d., 360 μm o.d.) was linear polyacrylamide (LPA) coated. Briefly, the capillary was flushed with 1 M sodium hydroxide, water, 1 M hydrochloric acid, water, and methanol in sequence. Then, the capillary was dried under nitrogen overnight. To introduce a double bond to the silica capillary wall, the capillary was incubated with 50% (v/v) 3-(trimethoxysilyl) propyl methacrylate in methanol at room temperature for over 24 h with both ends sealed by silica rubber. After that, the capillary was flushed with methanol and dried under nitrogen overnight. We prepared a solution of 4% (w/v) acrylamide in water and a solution of 5% (w/v) ammonium persulfate (APS) in water and mixed the two solutions by a 100:1 volume ratio. The mixed solution was degassed under nitrogen for 15 min and then introduced into the pretreated capillary by vacuum. Both ends of the filled capillary were sealed with silica rubber, followed by incubating at 50°C for 1 h to coat the capillary inner wall with LPA. The capillary was finally flushed with water to remove the residue reactant and stored at room temperature before use. To reduce the outer diameter of the capillary, the HF etching was operated for the separation capillary with the same protocol as the reference [29].

### 2.5 ∣ CZE-MS/MS

CZE-MS/MS platform was built up by connecting a CESI 8000 Plus CE system (Sciex) to Q Exactive HF mass spectrometer (Thermo Fisher Scientific) with a third-generation electro-kinetically pumped sheath flow CE-MS interface (an EMASS-II CE-MS interface, CMP Scientific) [30, 31]. An electrospray emitter with an orifice size ranging 25-35 μm was pulled with a Sutter P-1000 flaming/brown micropipette puller.

The background electrolyte (BGE) was 5% (v/v) acetic acid (AA, pH 2.4), with the sheath liquid consisting of 0.2% (v/v) formic acid and 10% (v/v) methanol in water. The etched end of the LPA-coated capillary was threaded through the tee of the CE-MS interface and introduced to the electrospray emitter. The electrospray voltage was set at 2.0 kV to the sheath liquid for electrospray ionization.

For the histone sample without gel fractionation, approximately 50 nL was injected. For the histone sample with gel fractionation, 50 or 100 nL of each fraction was injected. For CZE separation of the histone sample, 30 kV voltage was applied to the injection end for 1800 s, followed by 30 kV voltage and 10 psi pressure applied for 600 s to push out the residue analytes in the capillary.

The MS parameters were set as follows. For MS, we set the resolution as 120,000, the AGC target as 1e6, and maximum injection time (IT) as 50 ms. The scan range of MS was 400–1500. Only ions with charge states higher than 6 or undetermined were subjected to fragmentation. The top 5 most intense ions were isolated in the quadrupole with an isolation window of 2 m/z and subjected to fragmentation with the higher energy collisional induced dissociation (HCD) method. For MS/MS, the resolution, AGC target, and maximum IT were set as 60,000, 1e6 and 400 ms, respectively. Dynamic exclusion was set to 50 s.

### 2.6 ∣ RPLC-MS/MS

The histone samples were diluted in 0.1% (v/v) FA to 0.1 mg/mL for injection of 1 μL onto an EASY-RPLC^™^ 1200 system (Thermo Fisher Scientific). The solvent A used was 0.1% FA in water, and the solvent B was 0.1% FA in 80% ACN. The sample was directed onto the C4 analytical column (Sepax 3 μm 300 Å, column inner diameter 75 μm, outer diameter 360 μm, length 20 cm) at a flow rate of 0.3 μL/min. The gradient started at 5% B, going to 35% B in 10 min, then 35% B for 10 min, and it slowly ramped up to 65% B at 100 min. Then, solvent B was raised to 80% B for eluting all the remains on the column.

The MS data were acquired on a Q Exactive HF mass spectrometer (Thermo Fisher Scientific). The nanoelectrospray source was set to 2.2 kV, with the transfer tube set to 320°C and the RP lens set at 50%. MS1 spectra were acquired with the mass range of 400–1500 m/z, at 120k resolution (at m/z of 400), AGC target of 1E6, maximum injection time of 50 ms, and 3 microscans. Only ions with charge states higher than 6 or undetermined were subjected to fragmentation. The top 5 most intense ions were isolated in the quadrupole with an isolation window of 2 m/z and subjected to fragmentation with the higher energy collision dissociation (HCD) method. MS2 spectra were acquired at 60k resolution (at m/z of 400), AGC target 1E6, maximum injection time of 200 ms, and 3 microscans.

### 2.7 ∣ Data analysis

All the raw files were analyzed with Proteome Discoverer 2.2 software (Thermo Fisher Scientific) containing ProSightPD 1.1 node or TopPIC (Top-down mass spectrometry-based proteoform identification and characterization) software (version 1.6.3) [32]. For the Proteome Discoverer 2.2 software, the Bos taurus database was downloaded from http://proteinaceous.net/-database-warehouse-legacy/ (April 2018). The MS1 spectra were first deconvoluted using the “ProSight PD High/High cRAWler” node through the Xtract algorithm., The signal-to-noise ratio threshold, the lowest and the highest m/z were respectively set to 3, 200, and 4000 for both precursor and fragmentation Xtract parameters. For precursor and fragment ions, we set the mass tolerance as 2-Da and 10-ppm, respectively. To find unreported truncated proteoforms, a biomarker search was also performed, with 10 ppm tolerance for both MS1 and MS2. Furthermore, to match unexpected PTMs, the last search was performed with a 1000-Da mass tolerance for MS1 and a 10-ppm mass tolerance for MS2. The false discovery rate (FDR) threshold of 1% was applied to filter the proteoform-spectrum matches (PrSMs) and proteoforms.

For the TopPIC software, the MSConvert software [33] was applied to convert the raw files to mzML files, followed by the spectral deconvolution performed with the TopFD (Top-down mass spectrometry feature detection) software. The generated msalign files were subjected to TopPIC for database searching. The spectra were searched against a Bos taurus database from Swiss-UniProt (October 2023). Several PTMs were set as variable modifications, including phosphorylation (S/T/Y), oxidation (M), methylation/dimethylation/trimethylation (K/R), acetylation (K/R), acetylation on protein N-terminus, and citrullination (N/Q/R). The maximum number of variable PTMs was set as 5. The mass error tolerance was 15 ppm for precursor ions and fragment ions. A 1.2-Da mass tolerance was used to identify the PrSM clusters. The maximum mass shift was 500 Da, with the maximum number of mass shifts set to 0. FDRs were estimated using the target-decoy approach, with a 1% PrSM-level FDR and a 1% proteoform-level FDR employed to filter the identifications.

### 2.8 ∣ Experimental and predicted electrophoretic mobility ($\mu_{\text{ef}}$)

The calculation of the experimental and predicted $\mu_{\text{ef}}$ is based on the literature [16, 34, 35]. Equation (1) was performed to calculate experimental $\mu_{\text{ef}}$,

(1) $$\text{Experimental}\;\mu_{\text{ef}}=L∕((30-2)∕\;L\times t_{M})\;({\text{unit of cm}}^{2}kV^{-1}s^{-1})$$

where L is the capillary length in cm, 30 and 2 are separation voltage, and electrospray voltage in kV. The migration time $\left(t_{M},\;s\right)$ of each proteoform was obtained from the TopPIC database search results.

To calculate the predicted $\mu_{\text{ef}}$, Equation (2) was used,

(2) $$\text{Predicted}\;\mu_{\text{ef}}=\text{ln}\left(1+0.233\times Q\right)∕M^{0.411}$$

The Q is the number of charges of each histone proteoform in the liquid phase, calculated by the number of positively charged amino acid residues in the proteoform sequence (K, R, H, and N-terminus). M is the molecular mass reported by the search engine in Da.

## 3 ∣ RESULTS AND DISCUSSION

### 3.1 ∣ Optimization of CZE-MS/MS for histone proteoform identification

Due to the high similarity in structure of four core histones, H2A, H2B, H3, and H4, a highly efficient separation strategy is a prerequisite for histone proteoform identification. In this work, based on the separation orthogonality of SDS-PAGE and CZE, we developed a method integrating gel fractionation and CZE separation to improve the identification of histone proteoforms.

**FIGURE 1:**
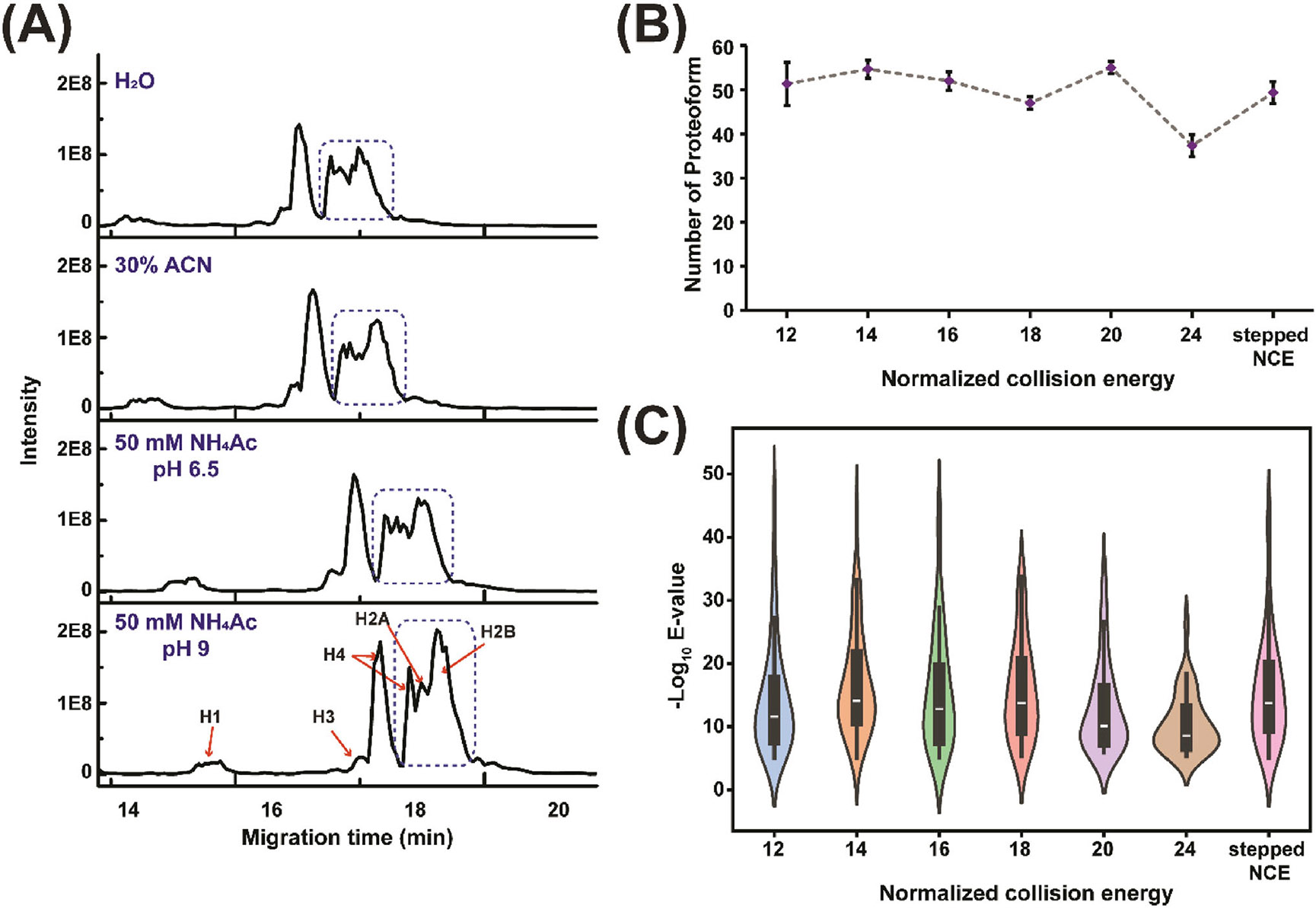
The optimization of sample buffers and normalized collision energy (NCE) of HCD for histone proteoform analysis with CZE-MS/MS followed by ProSightPD analysis. (A) The electropherograms of histone proteoforms with different sample buffers. With 50 mM NH4Ac (pH 9) as sample buffer, the histone proteins were resolved better than in other sample buffers (blue dashed circle). (B) The number and (C) E-value distribution of intact histone proteoforms identified with different NCEs. About 50 nL of sample was injected for each CZE-MS/MS run. The error bars represent the standard deviations of the number of proteoform identifications from technical triplicate measurements.

First, we tried to improve the separation of histone proteoforms with CZE. Two different online preconcentration methods, field-amplified sample stacking and dynamic pH junction methods, were employed for histone proteoform analysis. Dynamic pH junction-based CZE-MS/MS has been well recognized as a useful tool for TDP of complex proteomes [36, 37]. In this method, the protein sample is dissolved in a buffer with a much higher pH than the BGE of CZE (i.e., pH 6.5 vs. 2.4) and proteins are concentrated at the pH boundary before CZE separation. Two different sample buffers for dynamic pH junction were tested in this study, 50 mM ammonium acetate (NH_4_Ac) with pH 6.5 and 9. For the field-amplified sample stacking, H_2_O and 30% ACN in H_2_O were tested as the sample buffer. As shown in Figure 1A, all four different CZE conditions produced a similar level of separation of the histone proteins within 20 min. Histone H1 is well separated from the four core histone proteins and those four proteins are only partially separated (i.e., H3 and H4; H2A and H2B). The dynamic pH junction with the pH 9 sample buffer produced slightly slower overall electrophoretic mobility of histone proteoforms and slightly better separation resolution for H4, H2A, and H2B (blue dashed circle in Figure 1A).The sample buffer containing 50 mM NH_4_Ac with pH 9 was used in the following studies.

We subjected 50 ng of histone sample to a 40-min CZE-MS/MS analysis and 100 ng of histone sample to a 130-min RPLC-MS/MS analysis, respectively. The chromatogram from RPLC separation is similar to the literature data [38] (Figure S1A). The result was analyzed with ProSightPD and TopPIC softwares, respectively. Surprisingly, while there is no significant difference in the identified number from CZE-MS/MS using two software (118 vs. 126) (Tables S1 and S2), the RPLC-MS/MS produced 105 proteoform identifications with ProSightPD and 57 proteoforms with TopPIC software (Table S3 and S4). Interestingly, the TopPIC results showed that the RPLC-MS/MS identified a similar number of H3 proteoforms to CZE-MS/MS but identified much lower numbers of other histone proteoforms than CZE-MS/MS (Figure S1B). The overall trends of the CZE-MS/MS and RPLC-MS/MS data regarding the percentage of identified proteoforms are similar except that CZE-MS/MS tends to benefit the identification of H1 proteoforms. The data highlights that CZE-MS/MS is a valuable alternative to RPLC-MS/MS for TDP of histone proteoforms.

We further investigated how the normalization collision energy (NCE) of HCD impacts the characterization of histone proteoforms. We studied six different HCD NCEs, that is, 12, 14, 16, 18, 20, and 24, as well as one stepped method combining three different NCEs (14, 17, and 20). As shown in Figure 1B, there are no significant differences in the number of intact proteoform identifications with different NCEs except NCE 24, in which histone proteoforms could be overfragmented. We only considered the intact histone proteoforms without truncations here, leading to much smaller numbers compared to those mentioned in the previous paragraph. Figure 1C shows the −log (E-value) distribution of identified proteoforms under various NCE conditions. E-value indicates a nonlinear transformation of the number of matched fragment ions in an MS/MS spectrum. More matched fragment ions will produce a lower E-value, suggesting a high-confidence identification. When the NCE is too low or too high (12, 20, and 24), the median of the −log (E-value) is lower than other NCE conditions (14, 16, 18, and stepped), Figure 1C. This phenomenon is most likely due to insufficient or overfragmentation of histone proteoforms. Interestingly, we found that different HCD NCE tended to benefit the identification of proteoforms of different histone proteins, Table 1. Considering both the number of proteoform identifications and the E-values, optimal NCEs (highlighted in bold in Table 1) were determined for proteoforms of H1 (NCE 12), H2A (NCE 14), H2B (NCE 16), H3 (NCE 14 or stepped), and H4 (NCE 18 or stepped), respectively, and were used for the following measurements of proteoforms of corresponding histone proteins. For H3 and H4, the stepped NCE produced comparable or even slightly better results compared to NCE 14 (H3) and 18 (H4). To make the MS/MS method simple, we chose NCE 14 for H3 and NCE 18 for H4 in the following studies.

### 3.2 ∣ Multi-dimensional separations of histone proteoforms via combining SDS-PAGE fractionation with CZE-MS/MS

As shown in Figure 1A, CZE can only provide limited separation power for histone proteins, especially H2A, H2B, H3, and H4. It is necessary to develop multidimensional separation techniques to advance the separation of proteoforms of core histone proteins. We have coupled SEC and FAIMS to CZE-MS/MS for the top-down characterization of histone proteoforms with the production of more proteoform identifications compared to CZE-MS/MS alone [17, 16]. However, the resolution of SEC and FAIMS for the prefractionation of proteoforms is limited. In this work, we aim to combine SDS-PAGE-based prefractionation (PEPPI-MS) [19] to CZE-MS/MS to further advance the separation resolution of histone proteoforms. SDS-PAGE offers a high-resolution separation of intact proteins based on molecular weight.

**FIGURE 2:**
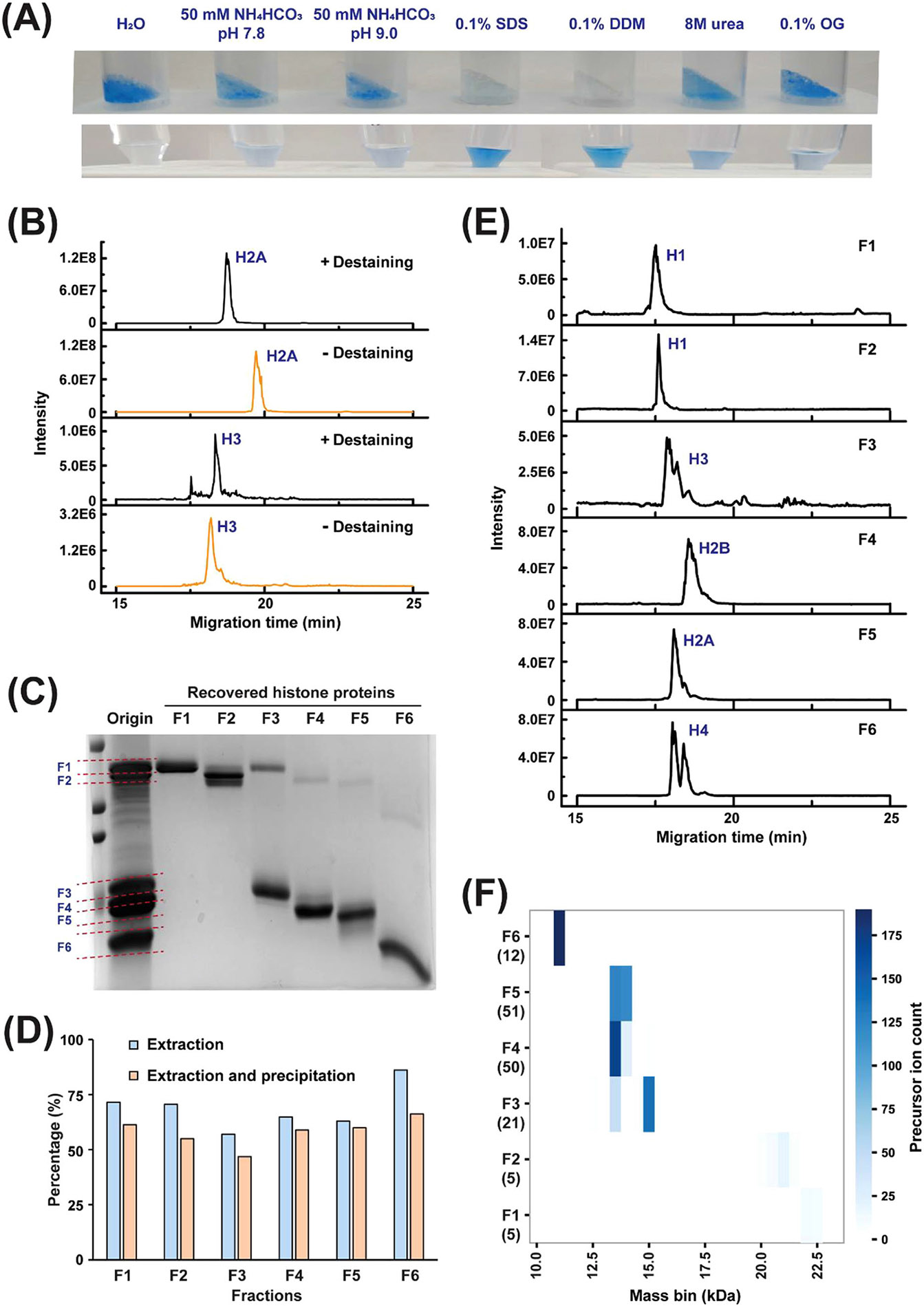
Optimization of PEPPI for histone protein fractionation. (A) Images of SDS-PAGE gel with stained proteins after histone protein extraction (top) and extracted protein solution (bottom) using different extractants, including H_2_O, 50 mM of NH_4_HCO_3_ (pH 7.8), 50 mM of NH_4_HCO_3_ (pH 9.0), as well as 0.1% DDM (w/v), 0.1% SDS, 0.1% OG and 8 M urea dissolved in 50 mM of NH_4_HCO_3_ (pH 9.0). (B) The electropherograms of H2A and H3 fractions recovered with (+) or without (−) destaining step. (C) Investigation of histone protein fractionation using the PEPPI workflow. Commercial calf histone proteins were separated by SDS-PAGE and stained with aqueous CBB. After dividing the sample lane into six portions (F1–F6), marked by dotted lines in the “Original” channel), proteins were extracted from each split gel using 0.1% SDS. Extracted proteins in each fraction were further separated by SDS-PAGE gels and shown in channels “Recovered histone proteins F1–F6.” (D) Recoveries for passive extraction. The recoveries for extraction and extraction followed by precipitation were estimated based on the intensity of each histone band. (E) The electropherograms of the individual histone protein fractions (F1–F6) recovered from SDS-PAGE gel. (F) Heat map depicting molecular weight distributions of precursor ions of histone proteoforms detected in each histone protein fraction. The precursor ion count represents the number of precursor ions with different masses detected in each protein fraction. The number in the brackets was from the TopPIC result and represents the intact proteoforms identified in each fraction.

For PEPPI-MS, separated proteins need to be extracted passively from the crushed gel and cleaned up before they are analyzed by CZE-MS/MS. We first optimized the protein extraction condition from gel for histone proteins. An 18% (the percent of acrylamide in the solution) separating gel was used to achieve a good resolution for the separation of histone proteins in the mass range of 10–25 kDa. Different extraction buffers, including H_2_O, 50 mM of NH_4_HCO_3_ (pH 7.8), 50 mM of NH_4_HCO_3_ (pH 9.0), as well as 0.1% n-dodecyl *β*-D-maltoside (DDM) (w/v), 0.1% SDS, 0.1% Octyl-beta-Glucoside (OG) and 8 M urea dissolved in 50 mM of NH_4_HCO_3_ (pH 9.0), were tested for histone protein extraction from the crushed gel. As shown in Figure 2A, the extracted protein solutions from SDS and DDM show the bluest color originating from the stained proteins, indicating SDS and DDM performed the best in histone protein extraction from the gel.

In addition, we compared different methods to purify the histone proteins from the extracted solutions containing SDS or DDM detergent, including trichloroacetic acid (TCA) precipitation, acetone precipitation, methanol/chloroform/water (M/C/W) precipitation, and gel filtration methods. The histone proteins purified with different methods were subjected to CZE-MS/MS analysis, respectively. As shown in Figure S2A, the M/C/W precipitation and gel filtration methods resulted in serious loss of some histone proteins. Interestingly, we found that the TCA precipitation method preferred the recovery of large H1 proteins while the acetone precipitation method preferred the recovery of small H4 proteins. With the acetone precipitation method under basic conditions, not only H1 could not be detected (Figure S2A), multiple satellite ions spaced +98 Da from the expected protein signal are also observed (Figure S2B), even with the rapid precipitation protocol. The phenomenon is most likely due to the artificial modifications introduced during the acetone precipitation step [39]. Therefore, the TCA precipitation method was employed for the subsequent experiments. The DDM-TCA and SDS-TCA produced similar results, Figure S2A. We chose SDS-TCA for all the following studies. Considering DDM is compatible with MS and has been widely used for native MS of membrane proteins [40], we expect that the DDM buffer could be useful for the native PEPPI-MS method to study protein complexes.

In the original PEPPI experiment [19], the destaining step is performed before MS analysis. In this work, we evaluated the histone protein recovery from gel with and without a distaining step. As shown in Figure 2B, while there is no significant difference for the highly abundant H2A protein with or without the destaining step, the destaining step resulted in a substantial sample loss for the low abundant H3 protein. Therefore, we processed the sample without the destaining step in the subsequent experiments.

With the aforementioned systematic investigations, our optimized PEPPI-MS workflow is that histone proteins are extracted from the gel using 0.1% SDS without destaining and cleaned up by TCA, followed by dynamic pH junction-based CZE-MS/MS analysis with 50 mM NH_4_Ac (pH 9) as the sample buffer.

We then tested the whole workflow. Twenty micrograms of histone proteins were separated by SDS-PAGE, and four main bands were observed, corresponding to H1, H3, H2A, and H2B, as well as H4, Figure 2C (origin channel). We then collected six fractions (F1–F6) from the SDS-PAGE gel according to their molecular weights, as marked by dotted lines. After extracted by 0.1% SDS and cleaned up by TCA, the histone proteins in each fraction were further characterized by SDS-PAGE (Figure 2C, Recovered histone proteins F1-F6 channels). As shown in Figure 2D, with the optimized PEPPI workflow, 57%–86% of histone proteins can be extracted from the gel, and the overall protein recovery rates from extraction and precipitation for all the fractions are about 50% or better, which is just slightly lower than the extraction step alone. Each protein fraction was redissolved in 50 mM NH_4_Ac (pH 9) and subjected to CZE-MS/MS analysis and searched with TopPIC software, with a total of 222 proteoforms identified (Table S5). As shown in Figure 2E, CZE-MS offered additional separations of proteoforms of each protein, especially H1, H3, and H4, which was further illustrated by the molecular weight distributions of the histone proteoforms detected in different SDS-PAGE fractions (Figures 2F and S3). With such a method, in comparison with CZE-MS/MS alone, not only 76% (222 vs. 126) and 122% (82 vs. 37) increase in the identification number of all proteoforms and intact ones were achieved, but also higher confidence in proteoform identification was obtained (Figure S4). The above results demonstrate the combination of SDS-PAGE and CZE-MS can be a highly useful technique for the top-down characterization of histone proteoforms.

We also subjected the fractions to the RPLC-MS/MS followed by TopPIC analysis, a total of 149 proteoforms were identified (Table S6), which is 33% less than those identified from a combination of SDS-PAGE followed by CZE separation. In addition, with semiempirical models that our group developed [16], the confidence of proteoforms identified from the CZE-MS/MS could be validated by examining the correlation between predicted and experimental $\mu_{\text{ef}}$ of proteoforms. The correlation of proteoforms identified from F6 was illustrated in Figure S5A, with a high correlation coefficient (R^2^ = 0.987) obtained for the unmodified histone proteoforms. Considering certain PTMs such as N-terminal acetylation and phosphorylation could reduce the charge (Q) of proteoforms by roughly one charge unit, the R^2^ between the predicted and experimental $\mu_{\text{ef}}$ of histone proteoforms increased from 0.945 (Figure S5B) to 0.957 (Figure S5C) after applying charge reduction for those acetylated and/or phosphorylated proteoforms, further improving the confidence of the histone PTM identification.

**FIGURE 3:**
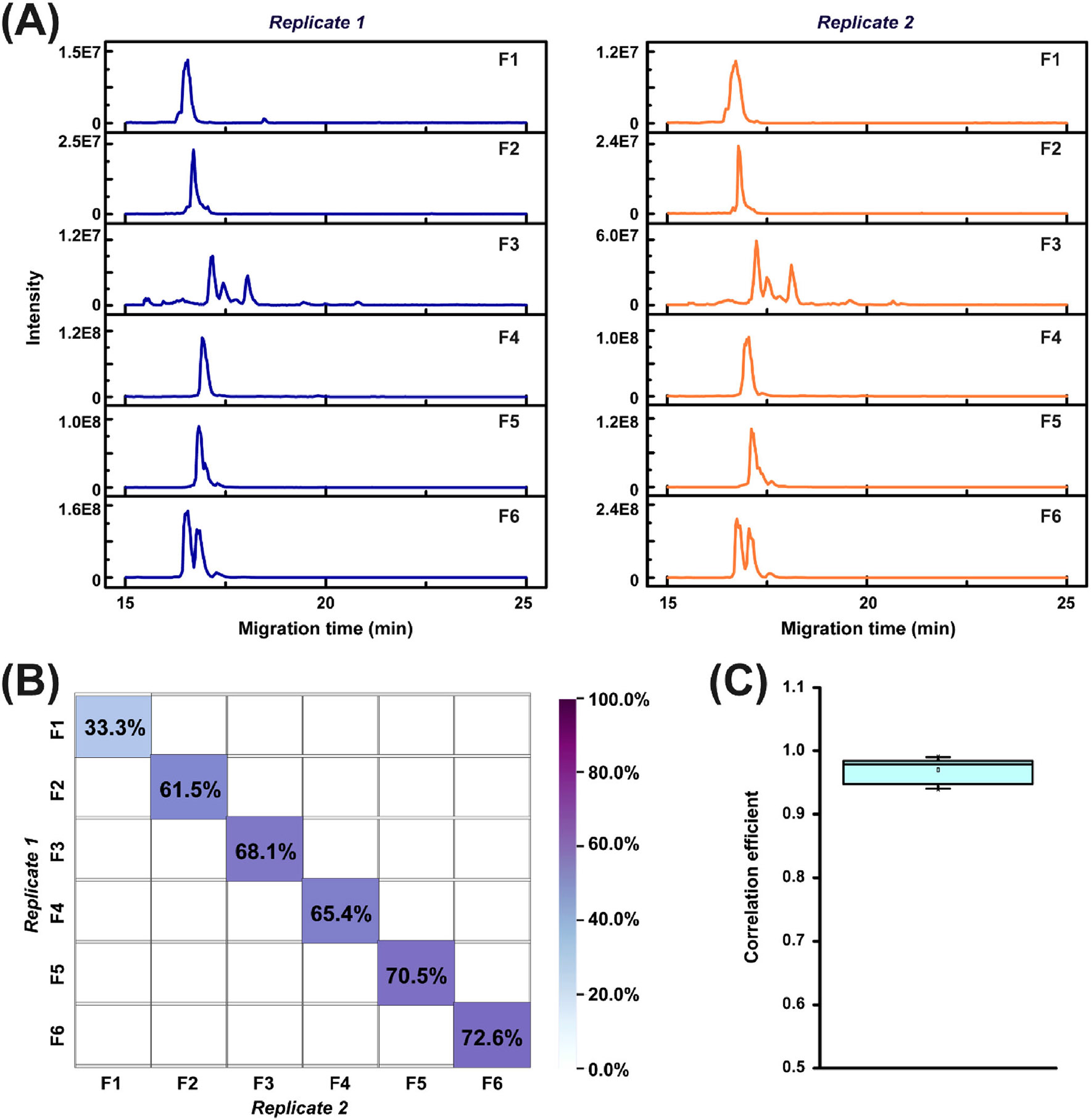
Summary of the reproducibility data of the SDS-PAGE-CZE-MS/MS followed by TopPIC analysis. (A) The electropherograms of different histone fractions of PEPPI (F1–F6) from two replicates. (B) Heat map depicting the overlap of detected histone proteoforms between each fraction of the two replicates. (C) Box-plot of correlation coefficient of proteoform intensity between the two replicates for the six PEPPI fractions.

We further evaluated the reproducibility of the SDS-PAGE-CZE-MS/MS method. Two aliquots of the histone sample were processed by the optimized PEPPI-MS and CZE-MS/MS conditions and are considered as two replicates (replicates 1 and 2), Figure 3. Briefly, each histone aliquot was separated by SDS-PAGE, and prepared by the optimized PEPPI-MS workflow, and each fraction was further analyzed by dynamic pH junction-based CZE-MS/MS in technical triplicate. Example electropherograms of each fraction for the two replicates are shown in Figure 3A. The consistent separation profiles between the same fractions of the two replicates indicate that the overall workflow is reproducible. Figure 3B shows the fraction-specific proteoform overlaps between the two replicates. Except for Fraction 1, 61.5%–72.6% of the proteoforms could be commonly identified by two replicates for each fraction. In addition, a high pairwise Pearson correlation coefficient of 0.94–0.99 for each fraction between two replicates was achieved (Figure 3C), further demonstrating the high reproducibility of the developed method.

### 3.3 ∣ Histone variants and PTMs

Histones are heavily post-translationally modified. Therefore, the ability to characterize the PTMs and their combinations on histones is vital to exploring the contribution of histone PTMs to the regulation of gene expression. In comparison with the truncated histone proteins, a more comprehensive characterization of distinct co-existing PTMs could be obtained at the intact histone protein level. As shown in Figure S6A, a relative wealth of fragment ions from intact histone protein could be produced in our study. Furthermore, the intact H2B histone variants that differ by a few amino acids near both the N- and C-termini were confidently identified (exemplified in Figure S6B).

**FIGURE 4:**
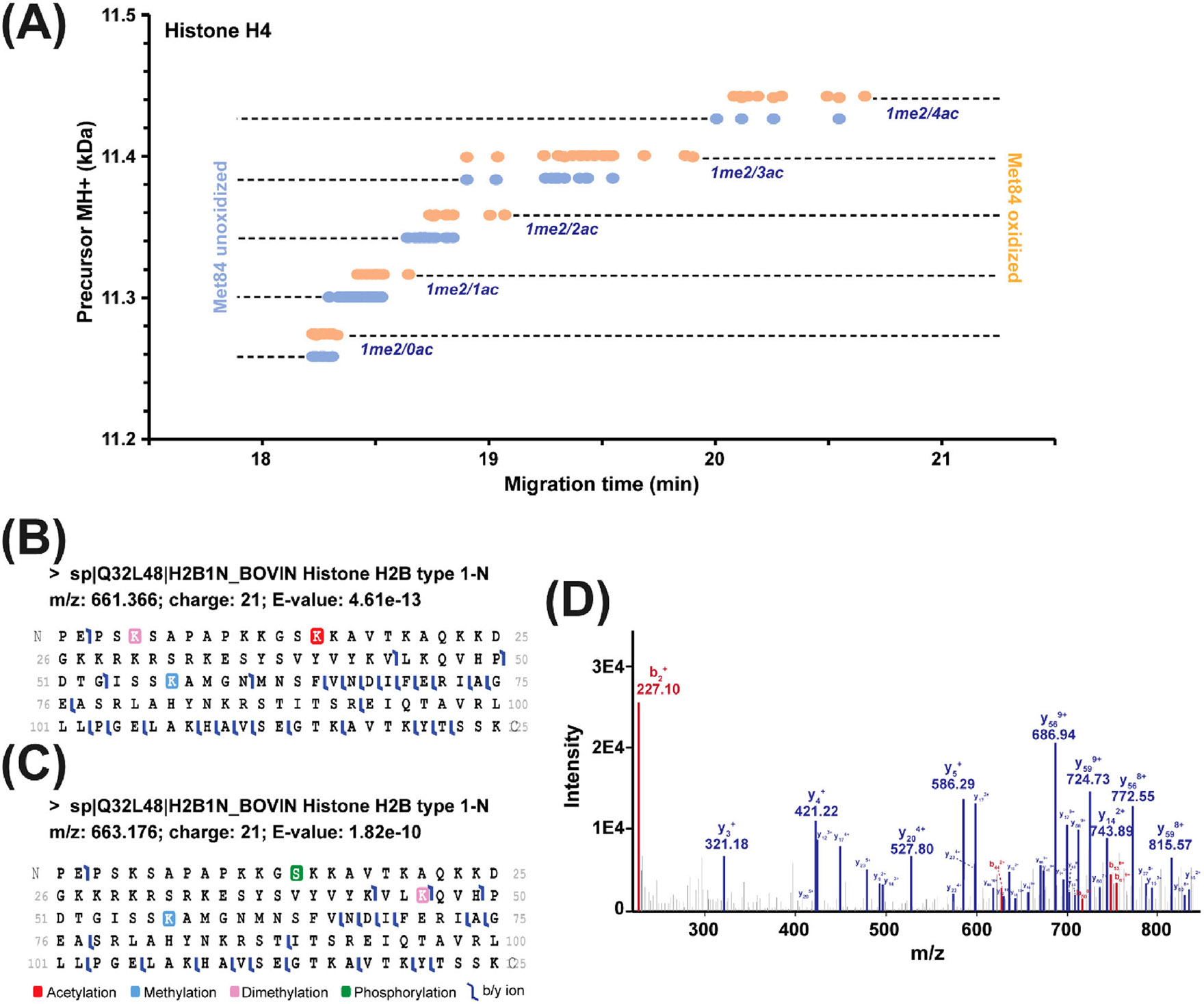
Summary of identified example histone proteoforms. (A) Histone H4 feature map of M84 oxidized and unoxidized proteoforms carrying one di-methylation and various numbers of acetylation from 0 to 4, which were separated by CZE-MS/MS followed by TopPIC analysis. (B) and (C) Sequences and fragmentation pattern of histone H2B type 1-N proteoforms containing different PTMs. (D) Tandem mass spectrum of the proteoform in (B) with fragment ion annotations. The x-axis shows the m/z values of the ions in the tandem mass spectrum. The data in (B)–(D) are from ProSightPD.

Here we also highlighted the value of our multidimensional platform for delineation of histone proteoforms carrying combinations of PTMs with some examples. Figure 4A shows the feature map of some histone H4 proteoforms with and without Methionine 84 oxidation as a function of CZE migration time. Those H4 proteoforms carried one di-methylation and various numbers of acetylation from 0 to 4. Interestingly, the H4 proteoforms carried more acetylation modifications migrated slower during CZE due to the significant positive charge reduction from the modifications [17, 35, 41]. The data clearly demonstrate that CZE is an extremely useful technique for separating histone proteoforms carrying PTMs that can significantly change the charge of proteoforms. Figure 4B,C shows two different proteoforms of histone H2B type 1-N identified with high confidence (E-values <1 × 10^−9^).The proteoform I (Figure 4B) had three PTMs, K57me, K5me2, and K15ac. Proteoform II carried K57me, S14ph, and K46me2.The annotated tandem mass spectrum of Proteoform I is shown in Figure 4D. Histone H2B phosphorylation at Serine 14 (H2B-S14ph) has been proposed as an apoptosis-specific marker of apoptotic cells [42, 43] whereas acetylation at the adjacent Lysine 15 (H2B-K15ac) is a property of nondying cells [44]. Our data is consistent with the previous discovery that the PTMs on the H2B are reciprocal, with deacetylation of H2B-K15 necessary to allow H2B-S14 phosphorylation [45].

We noted that the fragmentation coverage of those identified histone proteoforms is limited by HCD, which made the accurate localization of PTMs on histone proteoforms challenging. Indeed, much effort has been devoted to better confidence for history PTM localization. Brodbelt’s group applied 193 nm ultraviolet photodissociation (UVPD), for TDP of histones using a commercial calf histone sample [46]. Recently, they enhanced the histone protein characterization by combining UVPD and Proton Transfer Charge Reduction (PTCR) [47]. Jensen’s group employed electron capture dissociation (ECD) [48] to characterize the histone H3.1 N-terminal tail peptides. The combination of these efficient fragmentation methods and our developed separation strategy will allow the comprehensive characterization of histone proteoforms with combinations of PTMs.

## 4 ∣ CONCLUSIONS

We developed a novel two-dimensional approach for TDP of histone proteoforms via integration of SDS-PAGE and CZE-MS/MS. The new methodology produced reproducible and high-resolution separations of histone proteoforms, enabling the identification of over 200 histone proteoforms. SDS-PAGE offered efficient isolation and purification of each histone protein, that is, H1, H2, H3, and H4. CZE provided further separation of proteoforms of each histone protein based on their charge-to-size ratios, which could be influenced by the PTMs, for example, acetylation and phosphorylation. We expect that SDS-PAGE-CZE-MS/MS will be a useful tool for TDP of histone proteoforms extracted from complex biological samples.

The current methodology can be further improved in terms of separation resolution. For example, the separation resolution of SDS-PAGE for histone proteins could be improved via optimizing some critical parameters. CZE separation of histone proteoforms can be further advanced via employing a much longer separation capillary (i.e., 1.5 m) [49], applying a much higher separation voltage (i.e., 60 kV), and optimizing the composition of the BGE.

## Supplementary Material

supporting material 1

supporting material 2

## Figures and Tables

**TABLE 1:** Summary of the NCE data.

Histone		Normalization collision energy (NCE)						
		12	14	16	18	20	24	Stepped (14, 17, 20)
H1	# Proteoforms	**3 ± 1**	1 ± 1	0 ± 1	1 ± 1	0	0	0
	−log (E-value)	**7 ± 1**	7 ± 1	18 ± 0	6 ± 0	0	0	0
H2A	# Proteoforms	16 ± 3	**16 ± 2**	15 ± 1	11 ± 1	12 ± 2	9 ± 1	12 ± 1
	−log (E-value)	18 ± 2	**18 ± 3**	16 ± 1	17 ± 1	16 ± 1	13 ± 0	16 ± 0
H2B	# Proteoforms	6 ± 1	8 ± 1	**9 ± 1**	8 ± 1	9 ± 3	10 ± 3	9 ± 2
	−log (E-value)	21 ± 2	20 ± 2	**23 ± 4**	22 ± 1	18 ± 3	10 ± 0	22 ± 1
H3	# Proteoforms	24 ± 2	**24 ± 1**	23 ± 4	21 ± 2	26 ± 4	12 ± 1	**23 ± 4**
	−log (E-value)	13 ± 1	**13 ± 1**	13 ± 2	11 ± 1	9 ± 1	8 ± 0	**13 ± 1**
H4	# Proteoforms	2 ± 1	5 ± 1	5 ± 1	**6 ± 1**	8 ± 1	6 ± 1	**5 ± 1**
	−log (E-value)	18 ± 3	18 ± 2	17 ± 1	**19 ± 3**	17 ± 2	20 ± 1	**22 ± 2**

*Note*: The number of proteoform identifications and −log (E-value) of the identified proteoforms in different NCE conditions are shown as the median ± standard deviations from triplicate measurements. The optimized NCE condition for each histone protein is marked in bold. The data is from ProSightPD.

## Data Availability

The MS raw data have been deposited to the ProteomeXchange Consortium via the PRIDE partner [50] repository with the data set identifier PXD048061.

## Statement of significance

Comprehensive characterization of histone proteoforms carrying combinatorial post-translational modifications (PTMs) is fundamental for understanding their roles in modulating gene expression. Mass spectrometry (MS)-based top-down proteomics (TDP) is the ideal approach for this purpose. However, it requires high-resolution separation of histone proteoforms prior to MS and MS/MS. Multidimensional separations are essential because of the high complexity of histone proteoforms due to PTMs and sequence variations. Here, for the first time, we present a two-dimensional approach via combining SDS-PAGE to capillary zone electrophoresis (CZE)-MS/MS for the characterization of histone proteoforms. The technique enabled high-resolution and reproducible separation and characterization of histone proteoforms, identifying more than 200 histone proteoforms from a commercial calf thymus histone sample. We expect that the technique will substantially advance TDP analysis of histone proteoforms.

## Abbreviations:

BGE: background electrolyte
HCD: higher energy collision dissociation
NCE: normalization collision energy
PEPPI-MS: passively eluting proteins from polyacrylamide gels as intact species for mass spectrometry
TDP: top-down proteomics
