# Supplementary material for: Combining SDS-PAGE to capillary zone electrophoresis-tandem mass spectrometry for high-resolution top-down proteomics analysis of intact histone proteoforms
Source: Proteomics. Author manuscript; Available in PMC 2025 Sep 1. (PMC11647866; doi:10.1002/pmic.202300650)
Supplement: supporting material 1 [file NIHMS2041143-supplement-supporting_material_1.docx]

**Combining SDS-PAGE to capillary zone electrophoresis-tandem mass spectrometry for high-resolution top-down proteomics analysis of intact histone proteoforms**

Fei Fang, Guangyao Gao, Qianyi Wang, Qianjie Wang, Liangliang Sun^*^

**Affiliations**

Department of Chemistry, Michigan State University, 578 S Shaw Lane, East Lansing, MI 48824, United States.

* Corresponding author. [lsun@chemistry.msu.edu](mailto:lsun@chemistry.msu.edu)


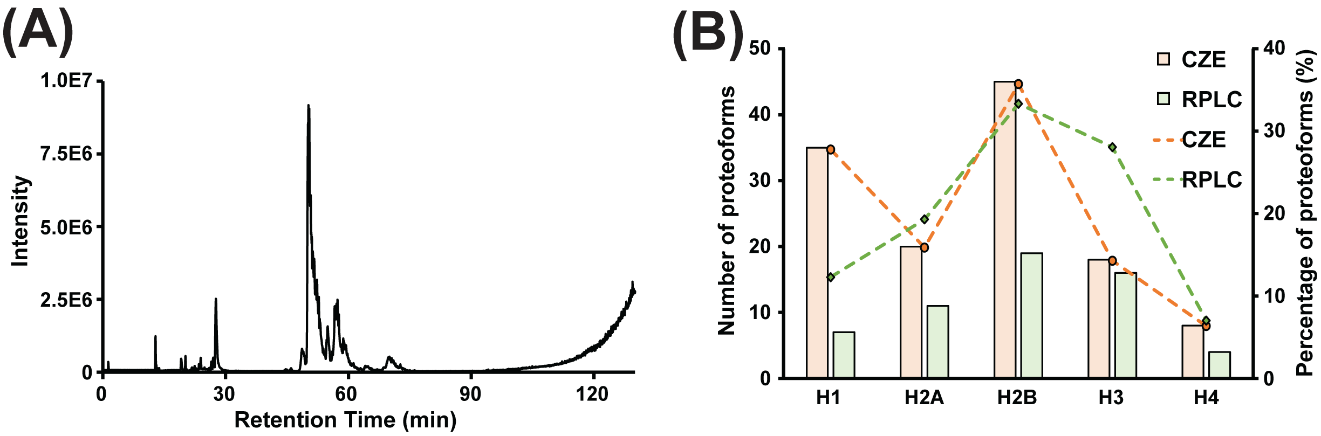


**Figure S1**. Comparison of CZE-MS/MS and RPLC-MS/MS for histone proteoforms identification with TopPIC database search. (A) The chromatogram of histone proteins separated by RPLC. (B) Comparison of histone proteoforms identified by CZE-MS/MS and RPLC-MS/MS. The bar represents the number of identified proteoforms and the line represents the percentage of identified proteoforms. The orange and green represent the proteoforms identified with CZE-MS/MS and RPLC-MS/MS, respectively.


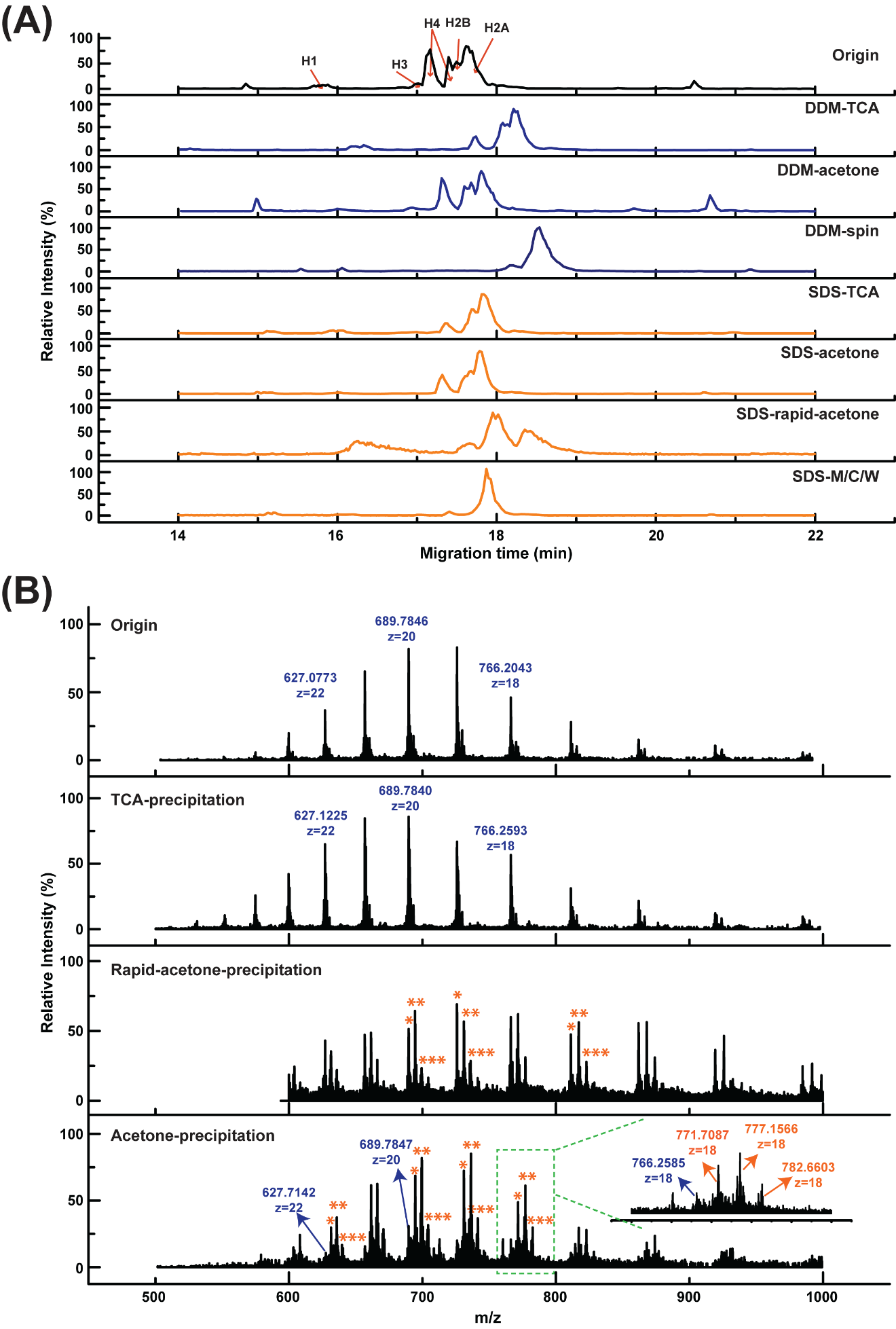


**Figure S2**. Different precipitation methods used for the histone protein purification from detergent solution. (A) The electropherograms of histone protein dissolved in 50 mM NH4Ac with pH 9 (origin), histone protein dissolved in 0.1% DDM (50 mM NH_4_Ac with pH 9) followed by TCA precipitation, acetone precipitation and gel filtration method, as well as the histone protein purified from 0.1% SDS (50 mM NH_4_Ac with pH 9) followed by TCA precipitation, acetone precipitation, rapid acetone precipitation and methanol/chloroform/water (M/C/W) precipitation method. (B) Deconvoluted mass spectra of H2B protein from histone protein sample dissolved in 50 mM NH_4_Ac with pH 9 (origin), histone proteins dissolved in detergent (0.1% SDS or 0.1% DDM) followed by TCA-precipitated, rapid-acetone-precipitation and acetone-precipitated. The peaks labeled by * in the acetone precipitation data represent proteins carrying one (*), two (**), and three (***) +98 Da modifications.


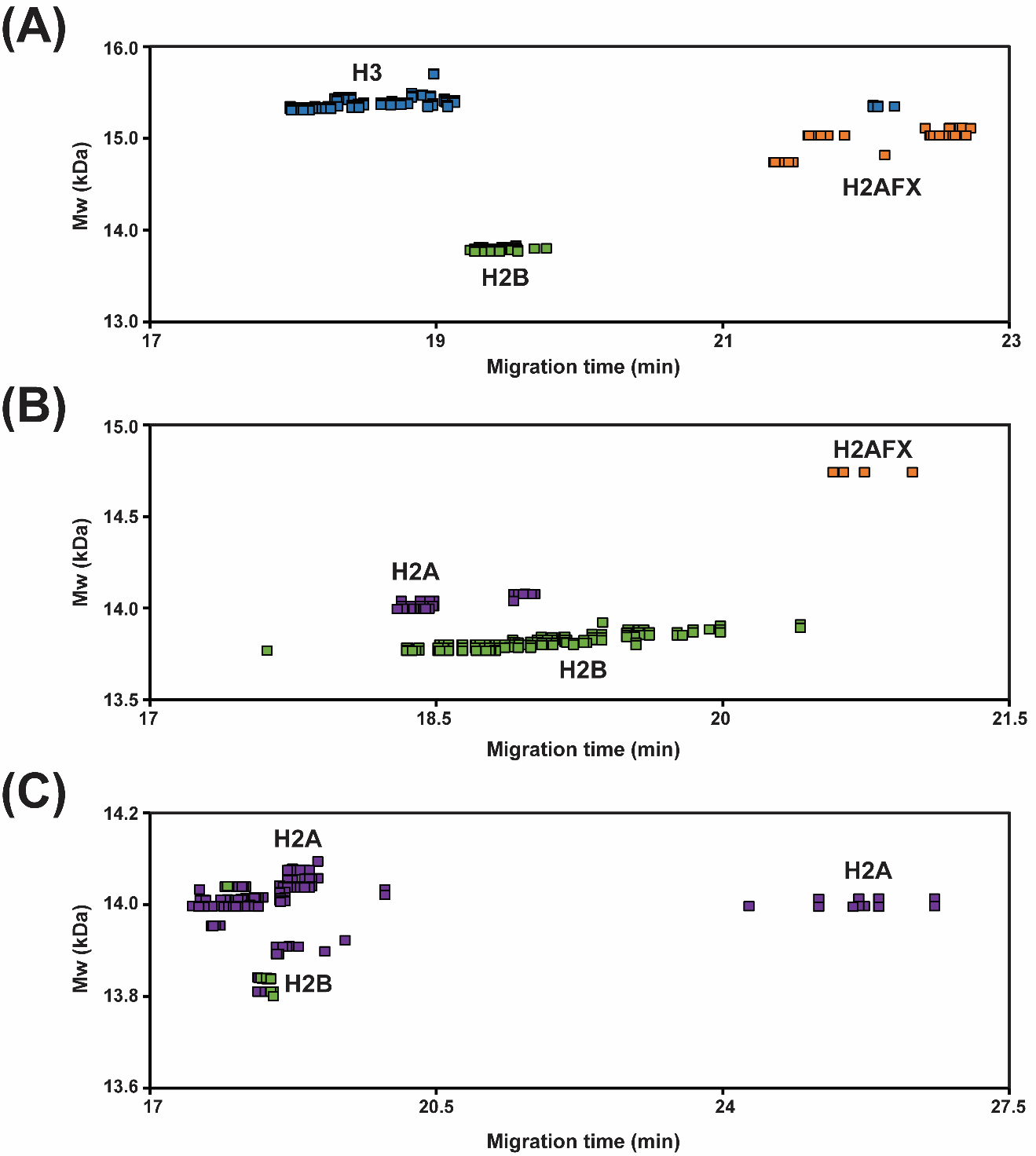


**Figure S3.** Histone proteoform separations with CZE-MS. Molecular weight (Mw) distributions of histone precursor ions identified in (A) fraction 3, (B) fraction 4, and (C) fraction 5. The square in blue represents H3, the square in orange represents H2AFX, the square in green represents H2B, and the square in purple represents H2A.


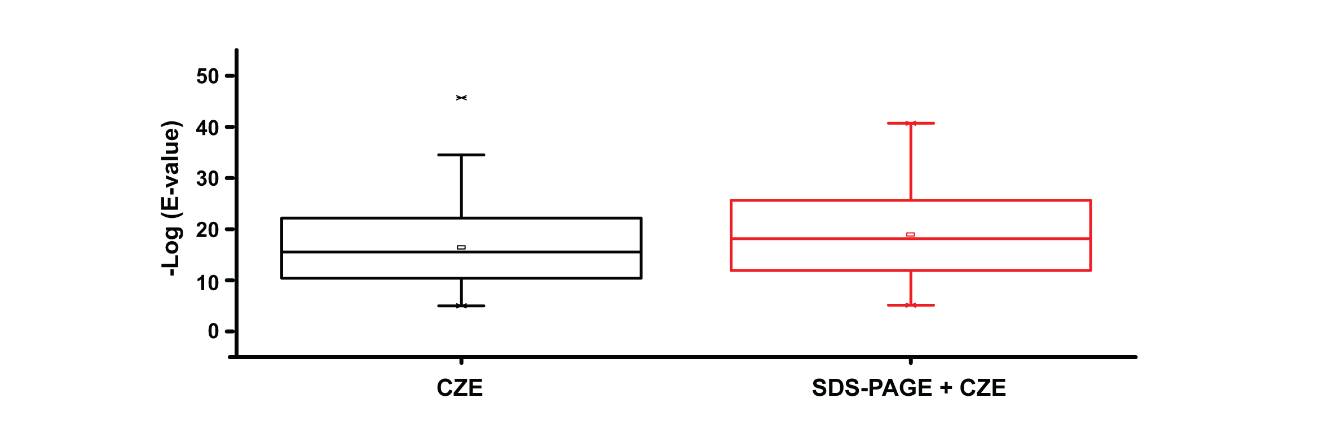


**Figure S4.** The confidence (E-value distribution) of the proteoforms identified from CZE method alone and SDS-PAGE combined with CZE method. The data is from TopPIC.


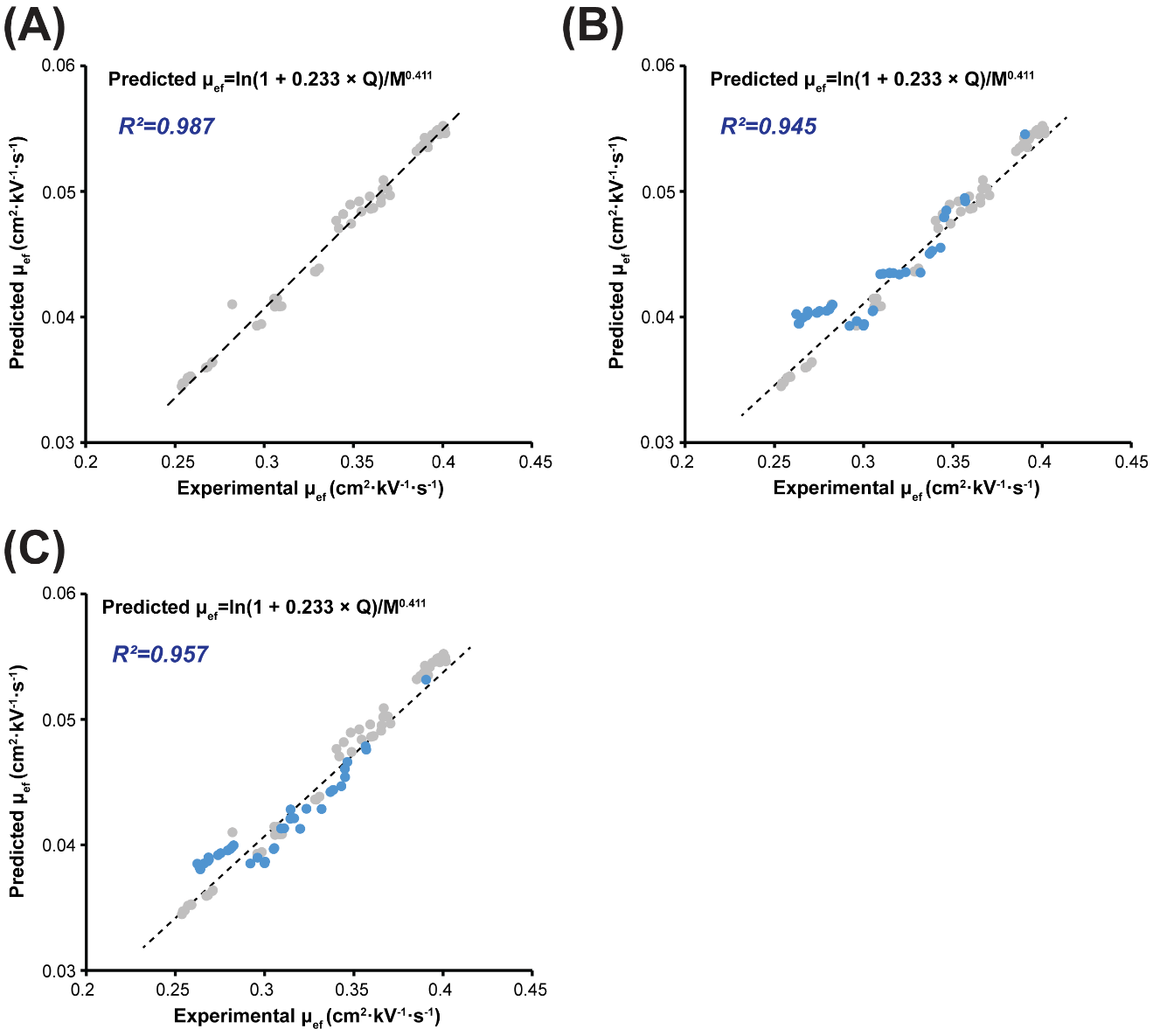


**Figure S5.** Predicting electrophoretic mobility µ_ef_ of histone proteoforms identified from CZE-MS/MS analysis under the BGE of 5% (v/v) acetic acid (pH 2.4). Linear correlations between theoretical and experimental µ_ef_ of (A) unmodified histone proteoforms, (B) histone proteoforms without charge correction, and (C) histone proteoforms with charge correction.


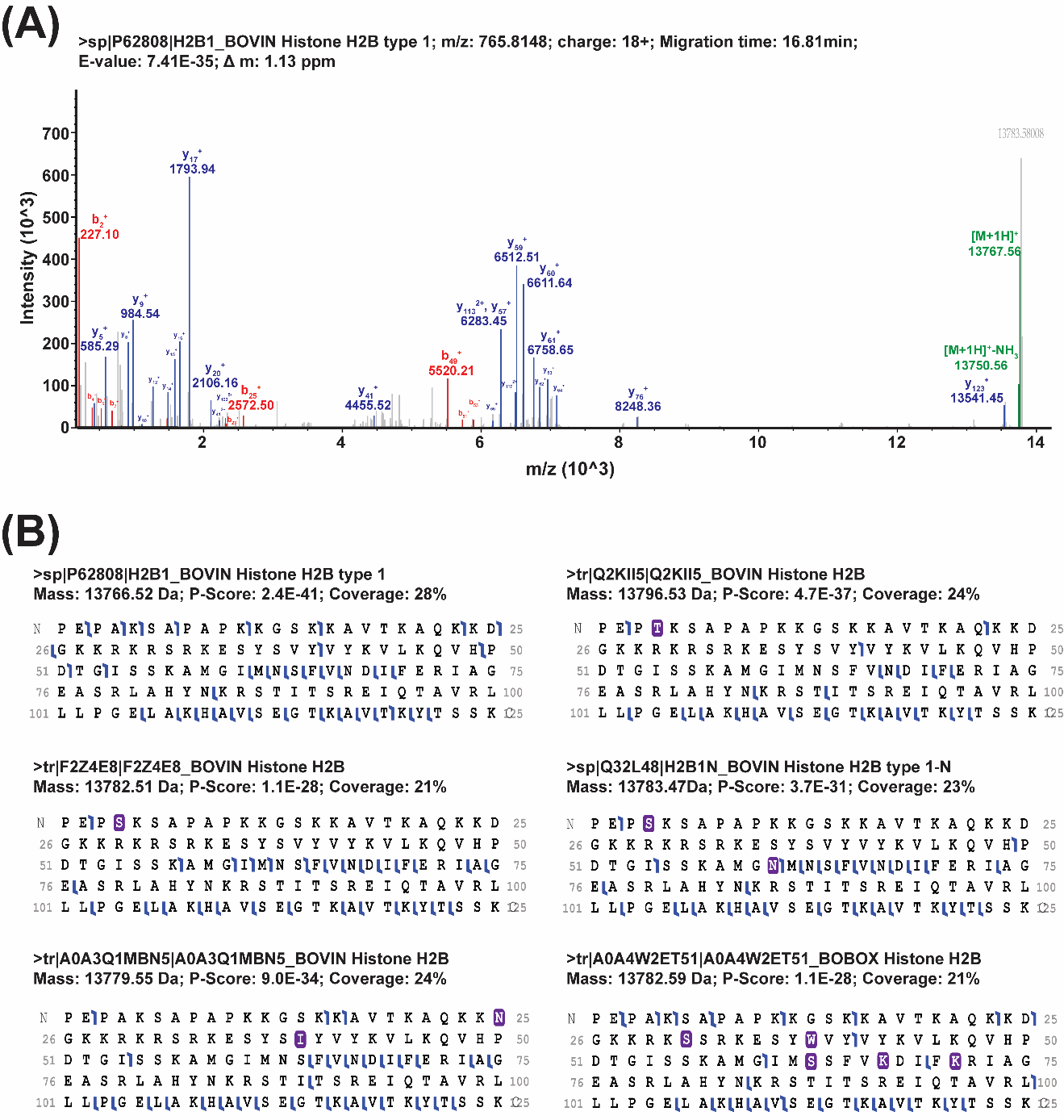


**Figure S6.** Fragmentation pattern and sequences of histone H2B variants from ProSightPD. (A) Fragmentation pattern of histone H2B type 1 (sp|P62808). (B) Sequences of histone H2B variants that differ by few amino acids near both the N- and C-termini. The amino acids marked in white are different from histone H2B type 1 sequence (sp|P62808).
